# Molecular Mechanisms Underlying Renin-Angiotensin-Aldosterone System Mediated Regulation of BK Channels

**DOI:** 10.3389/fphys.2017.00698

**Published:** 2017-09-13

**Authors:** Zhen-Ye Zhang, Ling-Ling Qian, Ru-Xing Wang

**Affiliations:** Department of Cardiology, Wuxi People's Hospital Affiliated to Nanjing Medical University Wuxi, China

**Keywords:** large-conductance calcium-activated potassium channels, renin, angiotensin ii, aldosterone, angiotensin-converting enzyme inhibitors

## Abstract

Large-conductance calcium-activated potassium channels (BK channels) belong to a family of Ca^2+^-sensitive voltage-dependent potassium channels and play a vital role in various physiological activities in the human body. The renin-angiotensin-aldosterone system is acknowledged as being vital in the body's hormone system and plays a fundamental role in the maintenance of water and electrolyte balance and blood pressure regulation. There is growing evidence that the renin-angiotensin-aldosterone system has profound influences on the expression and bioactivity of BK channels. In this review, we focus on the molecular mechanisms underlying the regulation of BK channels mediated by the renin-angiotensin-aldosterone system and its potential as a target for clinical drugs.

## Introduction

Large-conductance calcium-activated potassium channels (BK channels) are expressed on cell membranes and involved in diverse physiological processes including controlling membrane potential and Ca^2+^ homeostasis, setting vascular tone and blood pressure, controlling neuronal electrical activity and regulating endocrine hormone secretion. The renin-angiotensin-aldosterone system (RAAS) plays a key role in the maintenance of water and electrolyte balance and blood pressure regulation. In recent years, an increasing number of experiments have been conducted to explore the extremely complex relationship between BK channels and RAAS. This review provides an overview of the variety of mechanisms underlying the regulation of BK channels in different target tissues mediated by RAAS and its potential as a target for clinical drugs.

## Structural characteristics and physiological functions of BK channels

### Structural characteristics of BK channels

**Figure 1:**
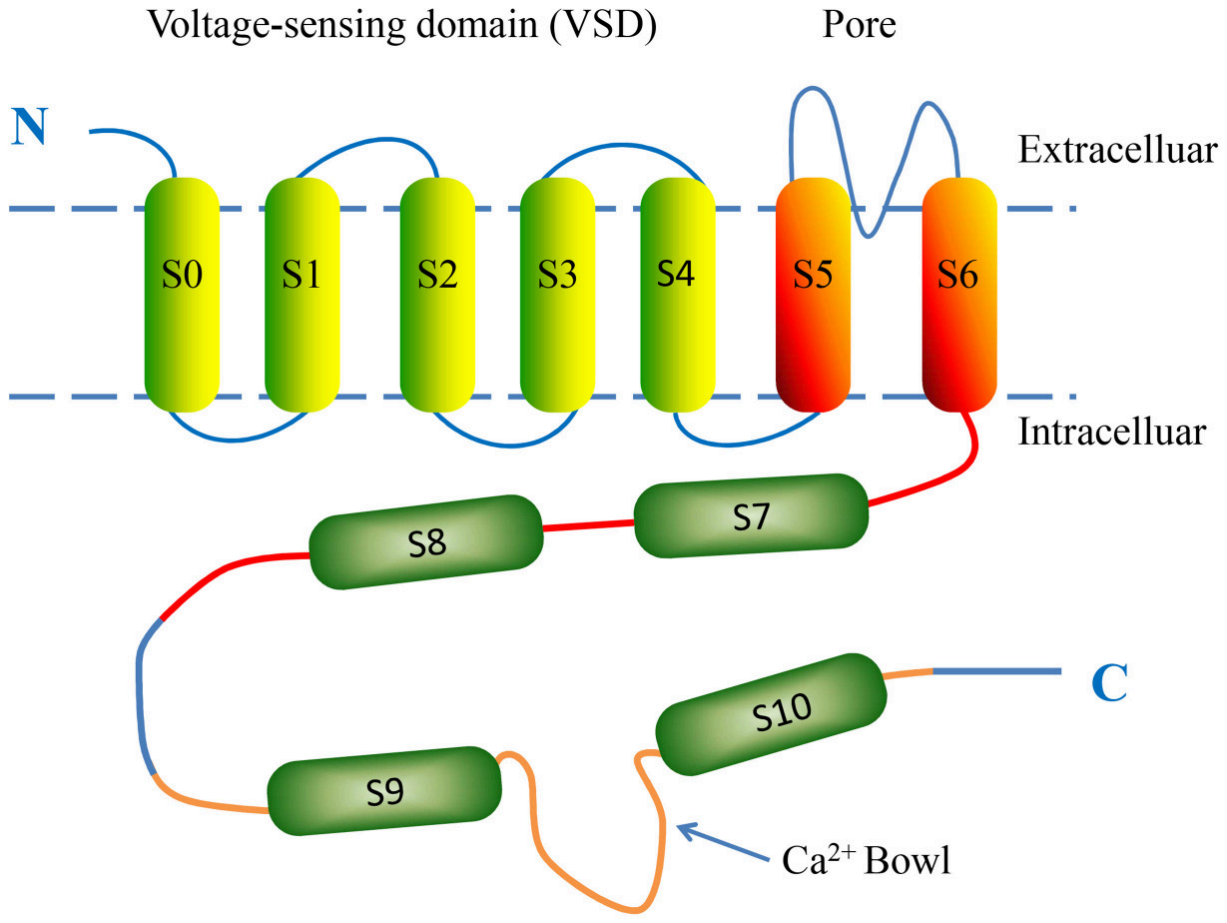
The putative structure of a single BK α-subunit. The α-subunit consists of seven putative transmembrane domains (S0-S6) and four hydrophobic regions (S7-S10). The voltage-sensing domain (VSD) is formed by four transmembrane helices S0-S4. Transmembrane helices S5 and S6 contribute to the K^+^-selective pore and selectivity filter. The intracellular C-terminal domain is comprised of two tandem structurally homologous regulator of conductance for K^+^ (RCK) domains, which possess BK intracellular ligand-binding sites. Regions S7-S8 and S9-S10 are located in the RCK1 (red) and RCK2 (orange) domains, respectively. The Ca^2+^ bowl (blue arrow) is a high-affinity Ca^2+^-binding site located in RCK2.

BK channels are widely expressed on various types of mammalian cell membranes. In some respects, BK channels are similar to the classic voltage-gated K^+^ channels, but differ in the mechanism of their activation (Salkoff et al., 2006). The minimal functional unit of the BK channel is a tetramer consisting of four α-subunits and auxiliary β-subunits (Lu et al., 2006). The α-subunit is encoded by *KCNMA1* and almost ubiquitously expressed. The α-subunit is formed by seven putative transmembrane domains (S0-S6) starting with an extracellular N-terminus and four hydrophobic regions (S7-S10) connecting with an intracellular C-terminus (Lee and Cui, 2010) (Figure 1). The BK channel voltage sensor domains (VSDs) are formed by the first four transmembrane helices (S1-S4) and sense changes in membrane potentials. Each α-subunit contains one VSD; four identical VSDs arrange counter-clockwise in a bundle and confer depolarization-dependent gating to the BK channel (Ma et al., 2006). S5 and S6, together with the intervening amino acids, form a K^+^-selective pore and selectivity filter, referred to as a pore-gate domain (PGD). S0 is a unique additional N-terminal transmembrane segment serving as a site of coupling with β-subunits; this functional interaction is responsible for modulating the balance between resting and active states of the VSD (Koval et al., 2007). The C-terminal region contains four helices (S7-S10) and makes up nearly two-thirds of the total length of the primary amino acid sequence. S9 and S10 are the most highly conversed among species and can be expressed as a separate domain. The cytosolic domain is comprised of two tandem structurally homologous but non-identical regulators of K^+^ conductance domains: RCK1 and RCK2 (Yusifov et al., 2010). Each RCK domain has its own high-affinity Ca^2+^ sensing site. One is located in the RCK1 domain and includes the residue D367. The other is located in the RCK2 domain and functions as the high-affinity Ca^2+^ binding sites (Ca^2+^ bowl) (Yuan et al., 2010).

The β-subunits can be classified into four subtypes (β1-4) that are encoded by genes *KCNMB1-4* respectively. The β-subunits are composed of two transmembrane helices, TM1 and TM2, with a large extracellular loop between them. TM1 lies close to S1 and S2, while TM2 is close to S0 (Liu et al., 2008). The β-subunits can alter the voltage and Ca^2+^ sensitivity of the α-subunits and regulate the kinetics and pharmacological properties of BK channels (Behrens et al., 2000). Recently, the four γ-subunits (γ1-4) of BK channels have been discovered and each contains a single-transmembrane domain. All four γ-subunits increase the voltage sensitivity of the BK α-subunit in the absence of Ca^2+^ (Li and Yan, 2016).

### Physiological functions of BK channels

BK channels link chemical and electrical signaling through Ca^2+^-dependent voltage-operated activation (Pantazis and Olcese, 2016). Moreover, a wide range of intracellular ligands enable BK channels to be gated or modified (Piskorowski and Aldrich, 2006). BK channels are known to be widely distributed not only in the plasmalemma but also in the inner mitochondrial membrane and other organelles (Balderas et al., 2015) and exhibit different electrophysiological properties (Chen et al., 2005). BK channels seem to be critical regulators of vital physical functions such as blood flow, neurotransmission, urination and immunity.

In vascular smooth muscle cells, BK channels act as regulators of membrane potential and smooth muscle tone (Wang et al., 2012). With regards to their physiological activity, BK channels are viewed as negative-feedback regulators of electrical excitation (Sausbier et al., 2005). In neuronal cells, BK channels regulate brain physiology (Grunnet and Kaufmann, 2004), including shaping action potentials (Gorini et al., 2010), monitoring neurotransmitter homeostasis and signaling, and neurovascular coupling for the maintenance of vascular tone (Singh et al., 2016). In skeletal muscles, BK channels participate in modulating transmitter release by repolarization of the action potential (Maqoud et al., 2017). Tricarico et al. (2005) found that BK channels are involved in the Ca^2+^-dependent phenotype transition of the fibers, which contributes to the skeletal muscle fatigue. In renal tissue, BK channels are expressed in various kinds of renal tubular segments including distal convoluted tubule (Belfodil et al., 2003), connecting tubule (Frindt and Palmer, 2004), and cortical collecting duct. Woda et al. (2003) proposed that BK channels are involved in the regulation of K^+^ balance in the distal nephron and the cortical collecting duct. Moreover, BK channels are involved in other various body functions, including regulating circadian rhythm regulation (Whitt et al., 2016), urinary bladder function (Meredith et al., 2004), susceptibility to hearing damage (Pyott et al., 2007) and erectile dysfunction (Werner et al., 2005).

### Main constituents and physiological functions of RAAS

RAAS also referred to as the renin-angiotensin system (RAS), is a crucial hormone system that contributes to maintaining water-electrolyte balance and regulating arterial blood pressure. BK channels are known to associate with several RAAS factors, with each of these interactions having distinct physiological consequences. The interaction between BK channels and RAAS is emerging as a key regulator of BK channel function in both health and disease.

Renin, a protein and enzyme generated from cells in the kidney, plays a critical role in RAAS. It can cleave and activate another circulating protein, angiotensin, which represents the beginning of a chain reaction involving RAAS.

Ang II, an octapeptide hormone, plays a major role in regulating the body's blood pressure and maintaining water-electrolyte balance through sympathetic nervous activity, aldosterone-regulated sodium excretion, and thirst responses (King et al., 2013). Ang II contains two types of high-affinity plasma membrane receptors, AT1R and AT2R, and each binding respectively brings about a serious of physical effects (Carey et al., 2000). Ang II-activated receptors generate and convert downstream signals to multiple intricate reactions.

Aldosterone, the main mineralocorticoid hormone, is involved in regulating sodium and potassium homeostasis and body fluid equilibrium by means of influencing the re-absorption of Na^+^ and excretion of K^+^ in the kidney (Balakumar et al., 2017). Aldosterone is traditionally thought to play a key role in regulating salt re-absorption and potassium secretion in response to two apparently opposite conditions: hypovolemia and hyperkalemia (Ruilope and Tamargo, 2017). With regards to the decrease in blood volume, aldosterone is released and causes Na^+^ retention via salt transport mechanisms in the distal nephron, while K^+^ secretion remains unchanged. In other words, water and sodium are retained without losing K^+^. With regards to the increase of plasma K^+^, aldosterone is also activated and promotes K^+^ secretion in the distal nephron, while salt re-absorption remains unchanged. This contradictory phenomenon is referred to as the aldosterone paradox (Ming, 2011). In both hypovolemia and hyperkalemia, aldosterone causes two distinct physiological effects in order to maintain the salt and K^+^ balance, which indicates that aldosterone-mediated regulation is dependent on multiple mechanisms (Arroyo et al., 2011).

### Regulation of BK channels mediated by RAAS

#### AT1R-activated signaling pathways

**Figure 2:**
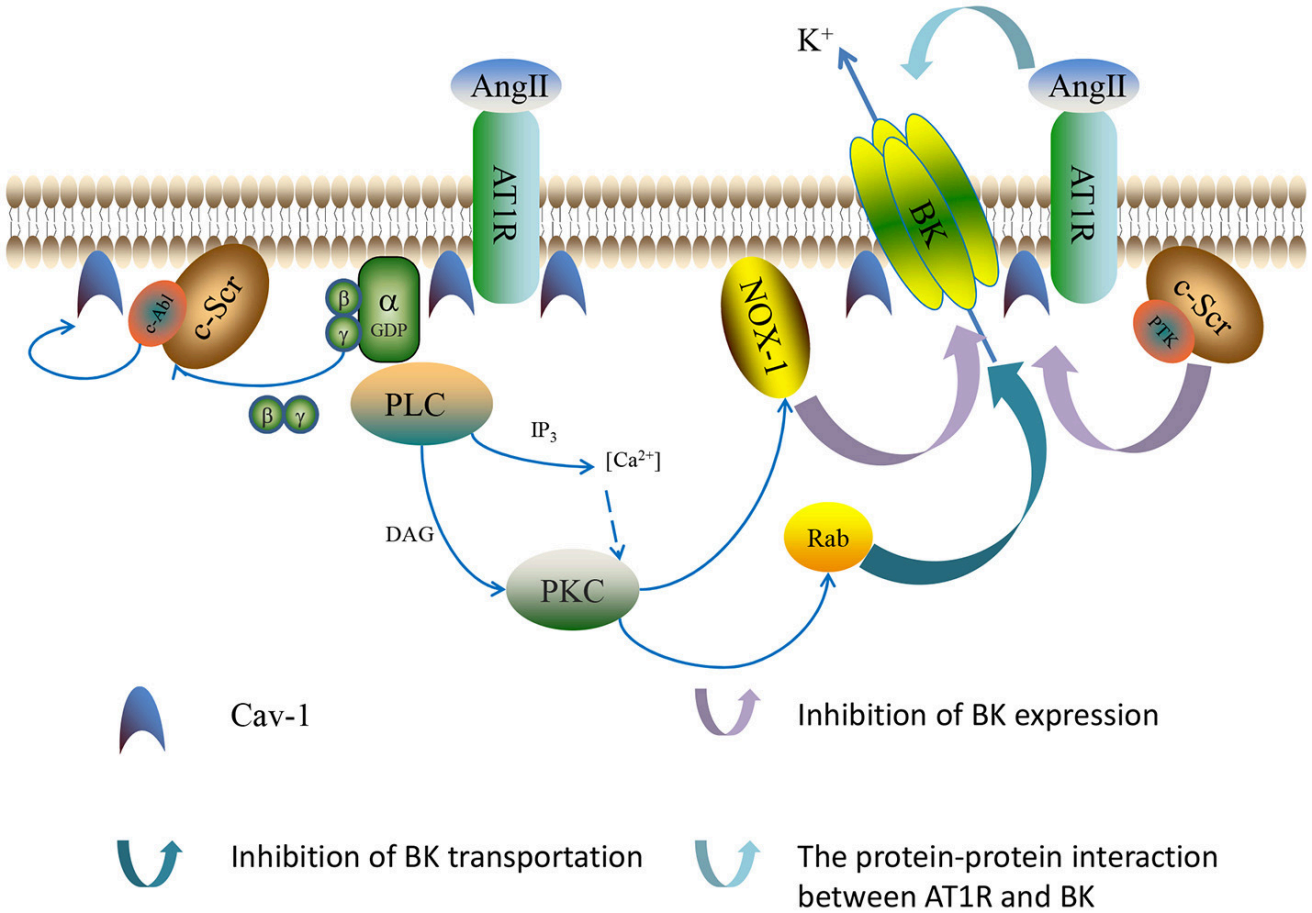
The mechanisms of Ang II-induced AT1R-dependent regulation on BK channels. AT1R interacts with Gα after binding with Ang II, which leads to the activation of the PKC and enhances NOX-1 complex activity. In turn, the released Gβγ activates the c-Src/c-Abl-dependent signaling pathway, leading to the tyrosine phosphorylation of Cav-1. ROS enhances c-Src/protein tyrosine kinase (PTK) activity, which results in an increase in protein tyrosine phosphorylation. PKC also inhibits the anterograde trafficking of BK channels by influencing the function of Rab-GTPases. In addition, AT1R and BK channels closely co-localize at the cell membrane and have a close protein-protein interaction.

An increasing number of studies apparently indicate that Ang II has an inhibitory influence on BK channels after binding to AT1R, whereas the molecular mechanisms are thought to be very complex and still unclear (Li et al., 2013). It has been reported that Ang II decreases the current amplitude of BK channels as well as the slope of the I-V curve and produces a left shift of the activation curves of BK channels in mouse podocyte cells. Ang II contributes to the pathogenesis of podocyte injury via the dysfunction of BK channels, which provides a theoretical basis for potential treatment of chronic glomerular disease in future (Gao et al., 2015). In recent years, the latest viewpoints have shown that Ang II-mediated inhibitive effects on BK channels are complicated, involving the redox-induced posttranslational modulation of BK α-subunits (Yoshimoto et al., 2004; Connelly et al., 2007), the regulation of BK β1-subunits expressions (Nieves-Cintrón et al., 2007), and a direct interaction between AT1R and BK α-subunit proteins (Zhang et al., 2014) (Figure 2).

#### G-protein-dependent signaling pathways

AT1R belongs to a family of seven transmembrane G protein-coupled receptors (GPCR) and regulate most of the physiological activities of Ang II in various kinds of target cells (Moslemi et al., 2016). AT1R plays a predominant role in the control of AngII-induced vascular functions. There are two AT1R subtypes in rodents, AT1_A_ and AT1_B_. G protein is a heterotrimer comprised of α, β, and γ subunits that enhances the combination and interaction between receptors and effector proteins. AT1R transmits signals through the activation of its downstream pathways that contains various kinds of signaling molecules (Burstein et al., 1997). There are two major downstream pathways stimulated by AT1R after binding with Ang II, consequently activating protein kinase C (PKC) and proto-oncogene tyrosine-protein kinase Src (c-Src) and leading to the activation of NADPH oxidase (NOX) (Thompson et al., 1998). NOX-1 and NOX-4 are the major NOX isoforms in human coronary arterial smooth muscle cells and have distinct subcellular distributions and regulation mechanisms (Hilenski et al., 2004). Caveolae-involved NOX-1 constitutes the major source of ROS generation in response to AT1R stimulation in vascular smooth muscle cells.

##### G-protein-involved signaling pathways

Gα-protein-mediated phospholipase C (PLC) phosphorylation is a primary mechanism through which Ang II exerts its function. The process leads to the activation of protein kinase C (PKC), a serine/threonine kinase (Touyz and Schiffrin, 2000). PKC consists of a multigene family of a few isoenzymes with diverse characteristics classified into three major groups. PKC is regarded as an important regulator of cellular functions (Wynne et al., 2009). A number of studies have demonstrated that PKC inhibits BK channels through various, complex mechanisms (Schubert et al., 1999; Xiao et al., 2014; Bo et al., 2015; Choi et al., 2015).

The expression of BK channels on the membrane is dependent on the balance of the dynamic change between transporting from the endoplasmic reticulum, recycling, endocytosis, and degradation (Conn and Ulloa-Aguirre, 2010; Simms and Zamponi, 2012). Rab-GTPases are known to control protein trafficking, including regulating vesicular transport and partaking in vesicle docking and fusion (Stenmark, 2009). The percentage of total β1-subunits localized on the plasma membrane is very small, and the intracullar β1-subunits are rich in mobile rab11A-positive recycling endosomes in smooth muscle cells (Leo et al., 2014). BK α-subunits are primarily transferred by rab4A-positive early endosomes to the plasma membrane. Leo et al. (2015) have found that PKC activated by Ang II inhibits BK channels not only by stimulating BK α-subunit degradation, but also by activating internalization and inhibiting anterograde trafficking of BK channels in cerebral artery myocytes. Experimental results of Leo et al. showed that the constriction to iberiotoxin (10 nM, IBTX) and dilation to NS-1619 (BK channel specific activator) (10 μM) of Ang II (100 nM, 8 h)-treated arteries were less than those in control untreated arteries, suggesting that Ang II stimulates BK channel internalization and degradation, leading to the construction of cerebral arteries. Recent studies demonstrate that the endocytosis and degradation of BK channel proteins are induced by glycosphingolipids or by PKC activation. The activation of PKC reduces channel recycling and diverts the channel to lysosomal degradation, which leads to a decrease in BK channel expression on the cell membrane. Ketsawatsomkron et al. (2012) demonstrated that the myogenic constriction is enhanced by PKC activation in endothelium denuded vessels. Zhou et al. (2010) have demonstrated that PKC-dependent phosphorylation of the α-subunit at S^1151^ and S^695^ in the channel pore is responsible for inhibiting BK channel function in HEK293 cells expressing recombinant channels. In addition, phosphorylation induced by PKC can also influence the stimulation of BK channel activity by protein kinase A (PKA) and protein kinase G (PKG), which results in the insensitivity of BK channels to the activation of PKG or PKA and subsequently inhibits cAMP-induced pulmonary vasodilatation (Barman et al., 2004). Hristov et al. (2014) have found that the reduction of BK channel activity in detrusor smooth muscle (DSM) is caused by PKC activation via a Ca^2+^-dependent mechanism, thus enhancing DSM contractility.

The Gβγ-subunits (Gβγ dimer), which form part of an inactivator of Gα subunits, play a significant role in AT1R-mediated regulation of BK channel activity. Gβγ activates c-Src, which in turn activates Abelson murine leukemia viral oncogene homolog (c-Abl) tyrosine kinase. C-Src, a non-receptor tyrosine kinase, is abundant in vascular tissues and can be activated by ROS as well, thereby forming a self-perpetuating activation loop and encouraging sustained generation of oxidative stress in response to Ang II stimulation. AT1R interacts with Gα after stimulation by Ang II, which in turn activates PKC and enhances NOX-1 complex activity through neutrophil cytosol factor 1 phosphorylation and Ras-related C3 botulinum toxin substrate 1 phosphorylation. In the meantime, the combination of Ang II and AT1R interacts with Gβγ, which activates c-Src/c-Abl-dependent signaling pathway. The process leads to the phosphorylation of Cav-1 tyrosine 14 mediated by c-Abl, which facilitates the trafficking of the receptor into relatively cav1-enriched lipid rafts and contributes to the Ang II-related BK channel dysfunction in diabetes. ROS not only increases c-Src/protein tyrosine kinase (PTK) activity but also inhibits protein tyrosine phosphatase (PTP) activity, which results in an increase of the phosphorylation in protein tyrosine. BK channel cysteine oxidation is directly caused by hydrogen peroxide (H_2_O_2_) and peroxynitrite (OONO^−^), the latter causing BK channel tyrosine nitration. Ang II-induced oxidative post-translational modification makes it possible to impair BK channel function in vascular smooth muscle (Ushio-Fukai and Alexander, 2006). In subsequent studies, Lu et al. (2016) proposed a molecular scheme of a receptor-enzyme-channel-caveolae microdomain complex and found that the colocalization between AT1R and BK α-subunits in vascular SMCs produces a physical disassociation between BK α and BK β1-subunits, enhancing Ang II-mediated inhibition of BK channels in STZ-induced diabetic rats. Caveolae-Ang II signaling is involved in vascular BK channel regulation and facilitates BK channel and coronary dysfunction in diabetes, indicating that BK channels may present a potential new clinical target in the prevention and treatment of cardiovascular complications in diabetes.

##### Roles of caveolae in Ang II signaling-dependent BK channel regulation

Caveolae are cell membrane flask-shaped invaginations and enriched in sphingomyelin and cholesterol, as a subset of lipid rafts. Caveolin, the signature protein of caveolae, is involved in the formation of caveolae and enabling caveolae to function as signaling organizing centers. Caveolin has emerged as an important scaffold protein that associates specific signaling complexes into caveolae and interacts with various kinds of signaling molecules (Krajewska and Maslowska, 2004). Among three mammalian isoforms of caveolin, caveolin-1 (Cav-1) is known to be a major structural protein and is widely expressed in vasculature (Cohen et al., 2004). Wang et al. (2005) have shown the co-localization of Cav-1 and BK channels in bovine aortic endothelial cells via immunofluorescence imaging. Moreover, a physical interaction between Cav-1 and BK channel proteins was confirmed by *in vitro* binding assay using GST-Cav-1 fusion proteins, showing that BK α-subunits directly interact with the Cav-1 scaffolding domains and producing an inhibitory effect on BK channel functions. In addition, the BK-Cav-1 interaction promotes the transition of the BK C-terminal end to the membrane and affects BK surface expression in native vascular tissue (Alioua et al., 2008).

Accumulating evidence suggests that caveolin and caveolae play a vital role in the regulation of AT1R-related and ROS-dependent Ang II signaling processes (Basset et al., 2009). Some studies demonstrate that Cav-1 is specifically involved in the ROS-dependent AT1R signaling events regulating SMC hypertrophy (Ushio-Fukai et al., 1998, 1999), which reveals the functional significance of Cav-1 in Ang II–mediated vascular pathophysiology and may provide new therapeutic strategies. Lu et al. (2010) have demonstrated that Ang II has an inhibitory effect on BK channels via Ang II-AT1R-caveolae oxidative stress signaling, which accelerates the development of vascular BK channel dysfunction in diabetes. Lu et al.'s experimental results have shown that Ang II-induced inhibition of BK channels was weakened in rat coronary vascular smooth muscle cells (SMC) with Cav-1 knock-down and in aortic SMC of Cav-1 knock-down mice, which indicates that Cav-1 plays an important role in this process. To determine the physiological relevance of these findings, the effects of Ang II and NS-1619 on the vasoreactivity of coronary arterial rings were measured and the results suggested that the components of IBTX-sensitivity BK channels and Ang II-induced vasoconstriction was reduced in STZ-induced diabetic rats, which may suggest an increased vascular tone in diabetic vessels. A recent study from same group has shown that genetic knockout of Cav-1 gene attenuated the myocardial infarction-induced by ischemia-reperfusion injury in diabetic mice (Lu et al., 2016). Such diabetic effects on myocardial infarction could be mimicked by 2 μM Ang II or 0.1 μM IBTX on the Langendorff-perfused hearts of non-diabetic wild type mice. Interestingly, 10 μM NS-1619 blocked the Ang II effects, but not IBTX effects, on myocardial infarction, suggesting that BK channels are the downstream target of Ang II signaling. Similar results were also found in the effects of forskolin on mouse myocardial infarct size reduction that was blocked by IBTX (Heinen et al., 2014). It is worthy to note that the protective effects of NS-1916 on myocardiac infarction are associated with mitochronfdral BK channnel activation (Sakamoto et al., 2008; Singh et al., 2013). Since IBTX is membrane impermeable, the authors argue that the vascular BK channel activation, at least in part, involves the protective roles of the experimental cardiac ischemia-reperfusion injury.

#### G-protein-independent signaling pathways

Ang II regulates various physiological effects by activating AT1R not only via a series of G-protein-dependent signaling pathways but also a number of G-protein-independent signaling cascades (Mehta and Griendling, 2007; Goette and Lendeckel, 2008). It has been reported that activation of calcineurin/the nuclear factor of activated T cells c3 (NFATc3) signaling pathway by Ang II downregulated BK β1-subunit expression in arterial smooth muscle cells, contributing to the BK channel dysfunction and the development of hypertension (Nieves-Cintrón et al., 2007). Recently, Zhang et al. (2014) have found that Ang II receptors and BK channels closely interact and co-localize at the cell membrane with high proximity indices in renal arterial smooth muscle. The Ang II-mediated regulation of BK channels is based on a close protein-protein interaction involving multiple BK regions. AT1R can diminish BK channel voltage sensitivity and inhibit BK activity independent of G-protein activation.

### AT2R-activated signaling pathways

AT2 receptors are highly expressed in fetal tissues and decreases rapidly after birth. They are much less abundant in adults and are found to be expressed in the pancreas, heart, kidney, adrenals, brain, and vasculature. The AT2R is a seven transmembrane G protein-coupled receptor and has 30% similarity with the AT1R. AT2R contributes to the regulation of renal, central nervous system and cardiovascular functions (De Gasparo et al., 2000). An increasing number of fundamental experiments indicate that AT2R may suppress AT1R-mediated biological effects due to triggering of activation of apoptosis via various signaling processes. Arima et al. (1997) reported that the activation of the AT2R may lead to endothelium-dependent vasodilation through a cytochrome P-450 pathway, which in turn opposes vasoconstriction induced by the AT1R in the rabbit glomerular afferent arteriole. Dimitropoulou et al. (2001) also demonstrated that the AT2R is able to enhance Ang II-induced endothelium-dependent vasodilation by stimulating BK channels in smooth muscle. The detailed mechanisms are still unknown and are expected to be further studied.

### Regulation of the hypovolemia state

Arroyo et al. (2011) proposed that Ang II plays a significant role in the distinct effects of aldosterone in hypovolemia and hyperkalemia, a process that involves WNK4 (Verissimo and Jordan, 2001). The WNK (with-no-lysine kinases) family is a serine/threonine kinase subfamily composed of four members known as WNK1 to WNK4, respectively encoded by genes *WNK1-4*. WNK1, WNK3, and WNK4 are primarily expressed in the kidney (Wilson et al., 2001). There is growing evidence that WNK4 functions as the “molecular switch” that alternates between different functional states to regulate Na^+^ and K^+^ transport in the distal nephron via mediating the activity of the Na^+^-Cl^−^ cotransporter (NCC) (Arroyo et al., 2011). Under conditions of hypovolemia (high Ang II and aldosterone levels), WNK4 demonstrates antagonistic effects of K^+^ secretion in the renal distal nephron.

The mechanisms underlying WNK4-induced inhibition of BK channels by down-regulating their expression are still not fully understood, and several different hypotheses have been put forward. Zhuang et al. (2011) proposed that WNK4 kinase is involved in K^+^ secretion in the renal distal nephron and inhibits BK channel activity dependent on its kinase activity. The inhibition likely results from lysosomal degradation instead of clathrin-mediated endocytosis of BK channels. Yue et al. (2013) drew a similar conclusion, proposing that WNK4-mediated inhibition of BK channels is due to the activation of ERK and p38 mitogen-activated protein kinase (MAPK) via a mechanism other than the enhancement of endocytosis in the cortical collecting duct. Wang et al. (2013) suggested that WNK4 or a WNK4 mutant with a region containing a coiled coil domain and a autoinhibitory domain may decrease BK α-subunit plasma membrane expression as well as the whole cell expression, which suggests that WNK4 inhibits BK channel function by enhancing the degradation of BK channels via an ubiquitin-dependent pathway in the distal nephron.

### Regulation of the high dietary potassium intake state

Diets rich in potassium can result in an increase in plasma potassium and promote the release of aldosterone. An increasing number of experiments have shown that, in condition of hyperkalemia, apical membrane expression of BK channels in the cortical collecting duct (CCD) is apparently elevated, which enhances K^+^ secretion and maintains K^+^ homeostasis (Najjar et al., 2005). However, there is considerable controversy on the issue as to whether or not the enhancement of BK channel activity is directly dependent on the high level of plasma aldosterone.

Some investigators hold the view that the increase in aldosterone may enhance the activity of BK channels under conditions of high dietary potassium intake. Cornelius et al. (2015) tend towards this view and demonstrated that high aldosterone concentration in low Na^+^, high K^+^ diet mice contributes to a high rate of BK channel-mediated potassium secretion, which leads to an osmotic diuresis. The high urinary flow created by BK-mediated K^+^ secretion may be activated in a positive feedback manner, indicating that BK channels may present a therapeutic target for treating hyperkalemia.

In contrast, some studies deem that aldosterone has little or no influence on the expression or the activity of BK channels in response to dietary potassium intake. Estilo et al. (2008) hold the similar view that renal K^+^ excretion is associated with a kaliuretic pathway, rather than aldosterone directly affecting renal potassium ion transport due to changes in plasma potassium concentration. Recent studies have revealed a new understanding of the major role of BK channels activated by aldosterone-independent kaliuretic factors in which BK channel expression levels are closely related with flow-stimulated K^+^ secretion (Woda et al., 2001; Welling, 2013). In the distal nephron, renal K^+^ excretion is deemed to be dependent on tubular flow rate and BK channels are activated by an increase in the tubular flow rate (Woda et al., 2003). Liu et al. (2007) proposed that flow-stimulated BK channel-mediated potassium secretion is related to a signal pathway that involves an increase in Ca^2+^ concentration, which enables BK channels to translocate from the cell to the apical membrane. Through further studies, they found that PKA and PKC are involved in regulating the activity of BK channels in the mammalian distal nephron (Liu et al., 2009).

## Effects of ACE inhibitors on BK channels

ACE inhibitors, which constitute a wide range of important clinical targeted drugs for RAAS, are a class of clinical drugs used mainly for the treatment of congestive heart failure and hypertension. Commonly prescribed ACE inhibitors include captopril, enalapril, zofenopril, perindopril, trandolapril, ramipril, and lisinopril. This group of drugs inhibits angiotensin-converting enzyme, an important component of the RAAS, which leads to hypovolemia as well as vasodilatation and causes decreased blood pressure and heart oxygen requirement. Albarwani et al. (2015) found that treatment with ACE inhibitor lisinopril increases the expression of BK channel proteins in mesenteric arteries. However, Li et al. (2014) obtained a contradictory result that the ACE inhibitor captopril fails to enhance the activity of BK channels. Captopril alleviates myocardial toxicity by reducing the generation of hemoglobin-based oxygen carriers (HBOCs), which is synergistically regulated by the reduction of NAD(P)H oxidase-induced ROS overproduction as well as increased NO bioavailability. The results of these studies show that the influences of captopril on BK channels are mediated by the endothelium instead of smooth muscle.

## Conclusion

In summary, RAAS is deemed to have great influence on the expression and bioactivity of BK channels to regulate its physiological function via multiple mechanisms mediated by different components in this system. Ang II possesses a dual-aspect function that acts on BK channels after binding with the two types of receptors, AT1R and AT2R. The aldosterone paradox is a recently proposed concept in which aldosterone leads to two distinct physiological effects under conditions of hypovolemia and hyperkalemia to maintain the fluid and electrolyte balance. To some extent, this indicates that aldosterone-mediated regulation of BK channels is a very complex biological process, rather than a straightforward one mediated by a single mechanism. ACE inhibitors, such as the clinical commonly used RAAS-targeted drugs, contribute to the beneficial effects mediated by the endothelium on the cardiovascular system. There are conflicting study results as to whether the process is related to the decreased activity of BK channels induced by ACE inhibitors. Hence, more in-depth studies are required in the future, with any novel findings applied to developing new clinical treatments.

## Author contributions

ZZ wrote the initial drafts. LQ and RW revised the review and finalized the last version of the article. All authors checked and approved the submitted version.

### Conflict of interest statement

The authors declare that the research was conducted in the absence of any commercial or financial relationships that could be construed as a potential conflict of interest.
